# Association between alleles, haplotypes, and amino acid variations in HLA class II genes and type 1 diabetes in Kuwaiti children

**DOI:** 10.3389/fimmu.2023.1238269

**Published:** 2023-08-10

**Authors:** Mohammed Dashti, Rasheeba Nizam, Sindhu Jacob, Hessa Al-Kandari, Ebaa Al Ozairi, Thangavel Alphonse Thanaraj, Fahd Al-Mulla

**Affiliations:** ^1^ Department of Genetics and Bioinformatics, Dasman Diabetes Institute, Dasman, Kuwait; ^2^ Department of Population Health, Dasman Diabetes Institute, Dasman, Kuwait; ^3^ Department of Pediatrics, Farwaniya Hospital, Ministry of Health, Sabah Al Nasser, Kuwait; ^4^ Clinical Care Research and Trials, Dasman Diabetes Institute, Dasman, Kuwait; ^5^ Faculty of Medicine, Kuwait University, Jabriya, Kuwait

**Keywords:** HLA, type 1 diabetes, HLA association and disease, amino acid variations, HLA alleles and haplotypes

## Abstract

Type 1 diabetes (T1D) is a complex autoimmune disorder that is highly prevalent globally. The interactions between genetic and environmental factors may trigger T1D in susceptible individuals. HLA genes play a significant role in T1D pathogenesis, and specific haplotypes are associated with an increased risk of developing the disease. Identifying risk haplotypes can greatly improve the genetic scoring for early diagnosis of T1D in difficult to rank subgroups. This study employed next-generation sequencing to evaluate the association between HLA class II alleles, haplotypes, and amino acids and T1D, by recruiting 95 children with T1D and 150 controls in the Kuwaiti population. Significant associations were identified for alleles at the HLA-DRB1, HLA-DQA1, and HLA-DQB1 loci, including DRB1*03:01:01, DQA1*05:01:01, and DQB1*02:01:01, which conferred high risk, and DRB1*11:04:01, DQA1*05:05:01, and DQB1*03:01:01, which were protective. The DRB1*03:01:01~DQA1*05:01:01~DQB1*02:01:01 haplotype was most strongly associated with the risk of developing T1D, while DRB1*11:04-DQA1*05:05-DQB1*03:01 was the only haplotype that rendered protection against T1D. We also identified 66 amino acid positions across the HLA-DRB1, HLA-DQA1, and HLA-DQB1 genes that were significantly associated with T1D, including novel associations. These results validate and extend our knowledge on the associations between HLA genes and T1D in Kuwaiti children. The identified risk alleles, haplotypes, and amino acid variations may influence disease development through effects on HLA structure and function and may allow early intervention via population-based screening efforts.

## Introduction

Type 1 diabetes (T1D) is a multifactorial autoimmune disorder, affecting over 8.7 million people worldwide and posing a major challenge to global healthcare systems (1). The aetiology of T1D is complex, involving a series of immunological and environmental factors that can trigger the disease in genetically susceptible individuals. The precise mechanism underlying β-cell destruction, leading to absolute deficiency of insulin and hyperglycaemia, is largely unknown. Hyperglycaemia develops after 80–90% of pancreatic β-cells are destroyed, providing a narrow window for therapeutic intervention (2). Insulitis is a major aspect of T1D pathogenesis, which is characterized by the infiltration of mononuclear cells, such as T cells, B cells, and macrophages, into pancreatic islet cells (3). T1D may lead to serious secondary complications involving neuropathy, nephropathy, and retinopathy (4); hence, early diagnosis is crucial in the treatment and management of the disease. Clinically, T1D is diagnosed by the presence of autoantibodies against pancreatic islet cells, including insulin autoantibodies (IAA), glutamic acid decarboxylase autoantibodies (GADA), islet antigen 2 autoantibodies (IA-2A), and zinc transporter 8 autoantibodies (ZnT8A) (5). Genetic predisposition to T1D has been evidenced by a positive family history and a heritability rate of over 50% in monozygotic twins (6).

The human leucocyte antigen (HLA) gene region, spanning a 7.6 Mb region on chromosome 6p21.3, is considered to be the strongest predictor of the disease, accounting for 40–50% of disease heritability (7). HLA class I and II genes are widely associated with several chronic debilitating autoimmune diseases, such as multiple sclerosis, lupus, thyroiditis, and T1D (8). Allelic and haplotypic combinations of three HLA genes, namely *DRB1*, *DQA1*, and *DQB1*, are widely associated with the development of T1D (7, 9). Allele-specific sequence motifs within the HLA-DQ and HLA-DR regions possibly determine the shape of the peptide binding groves and modulate T cell repertoire activity (8, 10). For instance, substitution of aspartic acid at amino acid position 57 of the HLA-DQ β chain tends to impart resistance, while replacement with non-Asp-57 has been associated with susceptibility to T1D in Caucasians (8, 11). Similarly, in individuals carrying the different HLA-DR4 subtypes, sequence variations at position β71 (engaged by glutamic acid/lysine/arginine), β74 (engaged by alanine/glutamic acid), and β86 (engaged by glycine/valine) lead to seven motifs (EAV, KAG, RAG, RAV, REG, REV, and KAV) that have a preferential impact on conferring resistance or susceptibility to T1D (10). According to the literature, multiple amino acid residues possibly impact the size and polarity of specific HLA anchor pockets and are likely to play a superior role in binding of autoantigen epitopes and presenting them T helper cells needed for specific islet autoantibody production, indicating its potential role in T1D pathogenesis (10, 12–15).

HLA class II haplotypes DRB1*03-DQB1*02:01 (DR3-DQ2) and DRB1*04-DQB1*03:02 (DR4-DQ8) are known to confer risk of developing T1D in multiple ethnic populations and are further associated with the development of β-cell autoantibodies (7, 16, 17). The DR3-DQ2 and DR4-DQ8 haplotypes, are present in 90% of Caucasian children with T1D (18, 19). Individuals with the HLA DR3-DQ2 haplotype are highly likely to produce GADA as their first autoantibodies, while those with the DR4-DQ8 haplotype preferentially present IAA in circulation (16, 17). An increased risk of T1D has been found in individuals carrying a heterozygous DR3-DQ2/DR4-DQ8 genotype compared with the risk associated homozygous DR3-DQ2/DR3-DQ2 and DR4-DQ8/DR4-DQ8 genotypes (20). On the contrary, certain haplotypes, such as DRB1*15:01*-*DQA1*01:02*-*DQB1*06:02, are reported to render protection against T1D, particularly in Caucasians (21).

The frequency and combinations of HLA alleles vary considerably across different ethnic populations. Identifying risk haplotypes is relevant in aiding clinical diagnosis of the disease and identifying individuals at risk of developing T1D. HLA haplotypes known to confer risk of developing T1D in the Japanese and Korean populations include DRB1*04:05-DQB1*04:01 (DR4-DQ4) and DRB1*09:01-DQB1*03:03 (DR9-DQ3); notably, a homozygous DR9-DQ3 haplotype revealed stronger susceptibility to T1D than a DR4-DQ4 haplotype (22). In sub-Saharan Africans, the HLA DR3 haplotype was reported to be more prevalent in individuals with T1D than the DR4 haplotype (23). HLA haplotype DRB1*07:01~DQA1*03:01~DQB1*02:02 was reported to confer susceptibility to T1D in African Americans, while the DQA1*02:01 allele in this haplotype tends to render protection against T1D in the European population (24, 25). HLA DRB1*03:01:01-DQB1*02:01 was reported to be the common locus that showed susceptibility to T1D in the Bahraini, Lebanese, and Tunisian individuals with T1D (26). A recent study conducted in the Emirati population showed a significant association between the DR3-DQ2 and DR4-DQ8 haplotypes and susceptibility to T1D. The highest risk of T1D was seen in individuals who carry the DRB1*03:01-DQB1*02:01/DRB1*04:02/05-DQB1*03:02 diplotype in Emiratis (27). While HLA class II alleles and haplotypes detected in individuals with T1D belonging to the Saudi Arabian and Omani populations were consistent to those in Caucasians and other Middle Eastern populations (28, 29).

As per the International Diabetes Federation (30), Kuwait ranks third among countries with an increased rate of incidence ofT1D (30). The incidence of T1D in children under the age of 14 years increased from 17.7 in 1992–1994 to 40.9 per 100,000 per year in 2011–2013 (31). Despite this, few studies have explored the impact of HLA variants on T1D pathogenesis (32, 33) in the Kuwaiti population. In the present study, we aimed to evaluate the association and contribution of HLA class II alleles with the risk of T1D in the paediatric Kuwaiti population using next generation sequencing. We intend to catalogue the entire spectrum of HLA class II alleles that impart susceptibility to, or render protection against, T1D.

## Materials and methods

### Ethics statement and study cohort

The study protocol was approved by the Ethical Review Committee of Dasman Diabetes Institute and was in accordance with the guidelines of the Declaration of Helsinki and the United States Federal Policy for the Protection of Human Subjects. The study cohort consisted of unrelated individuals with T1D (95) and controls (150). Participants with T1D were recruited from the registry initiated and maintained at Dasman Diabetes Institute, called the Childhood-Onset Diabetes eRegistry, which is based on the DiaMond protocol. The criteria for recruiting patients with T1D and information on participant consent are discussed in detail previously (31). The controls recruited in this study were non-diabetic individuals above 38 years of age.

### Targeted HLA data

For individuals with T1D, an Omixon Holotype HLA V3 kit (Omixon, Hungary) was used on genomic DNA (0.8–1.2 µg) extracted by the QiAmp DNA blood mini kit, following the manufacturer’s protocols. The HLA typing kit generated DNA libraries and sequences for 11 loci, and among them were the DQA1, DQB1, and DRB1 genes. The protocol involved long-range PCR amplification of HLA genes using locus-specific master mixes, followed by quantitation and normalization of the resulting PCR amplicon, using QuantiFlour dsDNA system (Promega, USA). Amplicons were then subjected to enzymatic fragmentation, were end repaired and adenylated, followed by index ligation. The resulting single pool of indexed libraries were selected using AMPure XP magnetic beads (Beckman Coulter, USA) and were quantified using the qubit fluorometer (Thermofisher Scientific, USA). Next-generation sequencing (NGS) was carried out on an Illumina Miseq (Illumina, USA) sequencer, following the manufacturer’s protocols.

### NGS exome data

For healthy controls, a Nextera Rapid Capture Exome kit (Illumina Inc. USA) was used on high quality genomics DNA for exome sequencing enrichment using an Illumina HiSeq 2500 platform (Illumina Inc. USA).

### HLA typing

Targeted and whole exome FastQ files were used as input for HLA-HD tool version 1.4.0 (34) to identify alleles in HLA class II genes (HLA-DRB1, HLA-DQA1, and HLA-DQB1*)* by comparing the reads to a reference panel from the IPD-IMGT/HLA database (35) version 3.46 (2021 October) build 2d19adf. The database can be accessed at https://www.ebi.ac.uk/ipd/imgt/hla/licence/.

### Testing for presence of celiac disease and Hashimoto’s thyroiditis

All T1D patients were tested for the presence of other comorbid conditions. Presence of celiac disease (CD) was tested using Anti-Tissue Transglutaminase: IgG, Anti-Tissue Transglutaminase: IgA (IU/ml) and Anti-Endomysial Ab (AEA) tests, while Hashimoto’s thyroiditis (HT) was tested using thyroid peroxidase antibody test.

### Statistical tests

Phenotype associations between haplotypes, alleles, and amino acids in HLA class II genes, including calculation of the Hardy-Weinberg equilibrium (HWE), confidence intervals (CI), odds ratios (OR), and P-values were analysed using Bridging Immunogenomic Data-Analysis Workflow Gaps (BIGDAWG) tool (36) on R console version 3.6.2 (https://www.R-project.org/). The associations between alleles and haplotypes were analysed based on high-resolution sequence-based HLA typing (3-field). In addition, alleles and haplotypes with low frequencies were combined into one group (binned) and discarded from the analysis. A P value of <0.05 was considered statistically significant. To adjust for multiple comparisons, Bonferroni correction was used where adjusted *P* < 0.05 (denoted as *P*c*) was considered statistically significant.

## Results

### Clinical characteristics

The average age of individuals with T1D (52 males and 43 females) was 13 years, with an average body mass index (BMI) of 21 kg/m^2^. Whereas the average age of healthy participants (50 males and 100 females) was 57 years, with an average BMI of 32 kg/m^2^. The age of onset in our T1D cohort was divided into 3 groups; <5 years old: 34%; 5–10 years old: 44%; and >10 years old: 22%.

### Comparison of HLA-DRB1, HLA-DQA1, and HLA-DQB1 allele frequencies between individuals with T1D and controls

The number of alleles identified in HLA-DRB1, HLA-DQA1, and HLA-DQB1 were 52, 21, and 40, respectively. All the identified alleles in HLA-DRB1, HLA-DQA1, and HLA-DQB1 passed the HWE test in participants with T1D and controls. Results of the associations between the three HLA class II genes among individuals with T1D and controls are shown in **Supplementary Table 1**.

The distribution of significant HLA-DRB1, HLA-DQA1, and HLA-DQB1 alleles between the T1D and control groups is shown in **Table 1**. A total of 51% of susceptible HLA-DRB1 alleles in the T1D group were from participants carrying the HLA-DRB1*03:01:01 and HLA-DRB1*04:05:01 alleles. In addition, a total of 41% of susceptible HLA-DQA1 alleles in the T1D group were from participants carrying the HLA-DQA1*05:01:01 allele. Furthermore, a total of 63% of susceptible HLA-DQB1 alleles in the T1D group were from participants carrying the HLA-DQB1*02:01:01 and HLA-DQB1*03:02:01 alleles. Whereas participants carrying HLA-DQA1*01:03:01 and HLA-DQA1*05:05:01 alleles contributed to a total of 23% of protective HLA-DQA1 alleles in the control group. Moreover, a total of 11% of protective HLA-DQB1 alleles in the control group were from participants carrying the HLA-DQB1*03:01:01 allele. Furthermore, only 5% of protective HLA-DRB1 alleles in the control group were from participants carrying the HLA-DRB1*11:04:01 allele. Nevertheless, to maximize the identification of significant alleles associated with T1D, 2 fields resolution analysis was performed where HLA-DQA1*03:01 allele [17% *vs*. 9% OR (95% CI) = 2.22 (1.23–4), *P*c = 0.04] passed the significance threshold and conferred risk to T1D.

**Table 1:** Distribution of significant DRB1, DQA1, DQB1 alleles in children with T1D and controls.

HLA gene	Allele	Frequency (%)		OR (95% CI)	*P*-value	*P*c*
		T1D	Controls			
DRB1	03:01:01	40	16	3.6 (2.29–5.63)	1.4 x 10^-9^	2.4 x 10^-8^
DRB1	04:05:01	11	3	4.0 (1.71–10.17)	0.00029	0.005
DRB1	11:04:01	0	5	0 (0–0.43)	0.00175	0.03
DQA1	01:03:01	1	10	0.1 (0.01–0.39)	9.4 x 10^-5^	0.001
DQA1	05:01:01	41	18	3.2 (2.05–4.92)	2.3 x 10^-8^	3.0 x 10^-7^
DQA1	05:05:01	2	13	0.1 (0.02–0.34)	7.4 x 10^-6^	9.7 x 10^-5^
DQB1	02:01:01	39	15	3.6 (2.3–5.66)	1.6 x 10^-9^	2.2 x 10^-8^
DQB1	03:01:01	2	11	0.1 (0.02–0.41)	6.8 x 10^-5^	0.0009
DQB1	03:02:01	24	9	3.1 (1.82–5.48)	7.7 x 10^-6^	0.0001

* Pc Bonferroni corrected p-value.

### Comparison of HLA-DRB1, HLA-DQA1, and HLA-DQB1 haplotype frequencies between children with T1D and controls

In total, we identified 100 unique DRB1~DQA1~DQB1 haplotypes. **Table 2** portrays results of the association between the DRB1~DQA1~DQB1 haplotypes. Haplotypes with few counts were binned as one haplotype. Two haplotypes conferred susceptibility to T1D; the most highly frequent and significant haplotype was HLA-DRB1*03:01:01~HLA-DQA1*05:01:01~HLA-DQB1*02:01:01 and the least frequent among controls but significant haplotype was HLA-DRB1*04:05:01~HLA-DQA1*03:03:01~HLA-DQB1*03:02:01. The HLA-DRB1*11:04:01~HLA-DQA1*05:05:01~HLA-DQB1*03:01:01 haplotype was more frequently expressed in controls, which may suggest its protective role against T1D; however, it is to be noted that it does not pass Bonferroni-corrected P-value though the OR is 0; thus, this allele cannot be considered as protective. Similarly, other well-known haplotypes were identified in our analysis, however it only shows significance in un-adjusted P values.

**Table 2:** Distribution of the DRB1~DQA1~DQB1 haplotypes among children with T1D and controls.

Haplotypes	Frequency (%)		OR (95% CI)	*P*-value	*P*c*
DRB1~DQA1~DQB1	T1D	Controls			
03:01:01~05:01:01~02:01:01	39	13	4.1 (2.61–6.63)	6.2 x 10^-11^	8.7 x 10^-10^
04:02:01~03:01:01~03:02:01	8	3	2.5 (1.02–6.32)	0.02	0.3
04:03:01~03:01:01~03:02:01	4	2	1.8 (0.57–6.06)	0.2	3.3
04:05:01~03:03:01~02:02:01	4	2	2.3 (0.61–9.14)	0.2	2.2
04:05:01~03:03:01~03:02:01	7	1	5.4 (1.64–23.16)	0.001	0.01
07:01:01~02:01:01~02:02:01	9	9	0.9 (0.49–1.95)	0.9	13.7
10:01:01~01:05:01~05:01:01	4	2	0.5 (0.12–1.74)	0.2	3.5
11:04:01~05:05:01~03:01:01	0	4	0 (0–0.55)	0.005	0.07
13:01:01~01:03:01~06:03:01	1	4	0.13 (0–0.87)	0.01	0.3
13:02:01~01:02:01~06:04:01	5	2	2.0 (0.68–6.69)	0.1	2.02
15:01:01~01:02:01~06:02:01	1	3	0.19 (0–1.46)	0.08	1.19
15:02:01~01:03:01~06:01:01	1	3	0.15 (0–1.1)	0.04	0.6
16:01:01~01:02:02~05:02:01	2	2	1.0 (0.22–4.51)	0.9	13.1
16:02:01~01:02:02~05:02:01	1	2	0.4 (0.04–2.38)	0.3	4.2

* Pc Bonferroni corrected p-value.

We examined the distribution of zygosity at two significant T1D risk HLA haplotypes across different age groups at onset. The first HLA haplotype, DRB1*03:01:01~DQA1*05:01:01~DQB1*02:01:01, had the highest percentage of homozygous individuals in the < 5 years age group at 11.7%, followed by 3.9% in the > 10 years group, and 0% in the 5-10 years group. The heterozygous individuals had the highest percentage in the 5-10 years age group at 22%, followed by 13% in the < 5 years group, and 6.5% in the > 10 years group. For the second haplotype, DRB1*04:05:01~DQA1*03:03:01~DQB1*03:02:01, the homozygous individuals had the highest percentage in the > 10 years age group at 1.3% and 0% in both the < 5 years and 5-10 years age groups. The Heterozygous individuals had the highest percentage in the < 5 years age group at 5.2%, followed by 2.6% in both the 5-10 years and > 10 years age groups.

### HLA-DRB1, HLA-DQA1, and HLA-DQB1 amino acid variation frequencies between children with T1D and controls

To enhance our comprehension of the aetiology of T1D, it may be helpful to investigate the mechanisms involved in the HLA class II genes molecules’ structure. Thus, we conducted a blind association analysis to assess the potential contribution of specific amino acid residues and their associations with T1D. After applying a Bonferroni correction to our analysis, we found that 66 amino acids positions were significantly associated with T1D, in which most comprised attributes related to protection and susceptibility, depending on the amino acid change (**Supplementary Table 2**). Of these residues, 16 amino acids on DRß1 (11, 13, 26, 33, 37, 58, 67, 70, 71, 73, 74, 96, 133, 140, 142, 180) were associated with T1D. The strongest association that conferred risk of T1D on the HLA-DRß1 positions were DRß1-Tyr-21, DRß1-Leu-67, DRß1-Lys-71, and DRß1-Arg-74 (*P*c = 6.4 x 10^-6^, *P*c = 3.6 x 10^-5^, *P*c = 0.0001, and *P*c = 0.0001, respectively). Additionally, among the 16 identified amino acids, DRß1-His-13, DRß1-Gln-70, DRß1-Lys-71, and DRß1-Arg-74 are already known to confer risk of T1D (10, 12, 29, 37, 38). Additionally, we identified a previously known amino acid position — DRß1-11 (29) — but with a different amino acid change. Of the total 66 amino acid positions, 21 positions identified at HLA-DQα1 (11, 18, 26, 41, 45, 47, 48, 50, 52, 53, 55, 56, 61, 64, 66, 76, 80, 130, 175, 187, and 218) were associated with T1D. Among them, a few were previously reported to be associated with the risk of T1D, such as DQα1-Arg-55 and DQα1-Arg-66 (38). Furthermore, of the total 66 amino acids positions, 29 were identified at HLA-DQβ1 (9, 13, 26, 28, 30, 37, 45, 46, 47, 52, 53, 55, 57, 66, 67, 70, 71, 74, 84, 85, 86, 87, 89, 90, 125, 167, 203, 220, 221), and were associated with T1D. Of these, DQβ1-Tyr-9, DQβ1-Leu-26, DQβ1-Ser-30, DQβ1-Phe-47, DQβ1-Ala-57, and DQβ1-Arg-70 were previously reported to confer risk of T1D (10, 14, 15, 37–39). Based on our analysis, the strongest association identified was from DQβ1-Ala-57, a position well-known to confer risk of T1D (39).

### Comorbidity with celiac disease and Hashimoto’s thyroiditis

Upon testing the T1D patients for the presence of celiac disease (CD) and Hashimoto’s thyroiditis (HT), we observed 2 individuals with CD and 3 individuals with HT.

CD was identified in a 10-year-old female child with T1D and HT. She belongs to a family with two siblings presented with a young onset age of 3 years for T1D. The patient is positive for Anti TPO antibodies (363.4 IU/ml), Anti-Endomysial Ab (AEA), Anti-Tissue Transglutaminase IgG (15.2 IU/ml) and Anti-Tissue Transglutaminase (IgA >200 IU/ml) tests. The second patient was a 6-year-old female child positive for Anti-Endomysial Ab (AEA) test indicating celiac disease, alongside T1D.

HT was confirmed in 3 female children aged less than 15 years, presenting T1D at a young age of less than 3 years. They confirmed HT diagnosis with an anti-TPO antibody level of >120.7 IU/ml.

Two out of the 3 HT patients and 1 out 2 CD patients carried the risk DRB1 03:01:01~ DQA1 05:01:01~ DQB1 02:01:01 haplotype, while in the other two patients no known risk haplotypes were detected.

## Discussion

The current study identified frequencies of significant alleles, haplotypes, and amino acid variants of major HLA class II genes between Kuwaiti children with T1D and controls.

When comparing the significantly associated alleles identified in our study within the HLA-DRB1, HLA-DQA1, and HLA-DQB1 genes with other Arab populations (non-Kuwaitis) and Caucasians with T1D, we found that most of our findings agree with those of previous studies (40; 7, 9). In regard to the Arab population, with the same genetic structure, we found that the most significantly associated allele out of the three alleles within the HLA-DRB1 gene was DRB1*03:01:01 [OR (95% CI) = 3.6 (2.29–5.63), *P*c = 2.4 x 10^-8^]. This gene confers risk of T1D both in our study and in neighbouring populations, such as the Saudi Arabian (28), Emirati (27), Bahraini (41, 42), Omani (29), Egyptian (43), Lebanese (42), and Tunisian (44, 45) populations. A previous association study carried out in the Kuwaiti population did not include the HLA-DRB1 locus (32); however, it is now known that a high number of Kuwaiti people with T1D carry the DR3 genotype (46). The second-most significant allele found in HLA-DRB1 was DRB1*04:05:01 [OR (95% CI) = 4.0 (1.71–10.17), *P*c = 0.005], which also confers risk of T1D in our study, along with the Saudi Arabian (28), Omani (29), Egyptian (43), and Tunisian (44, 45) populations. Moreover, a high number of Kuwaiti individuals with T1D have been found to carry the DR4 genotype (46). The third-most significant allele, DRB1*11:04:01, demonstrated resistance to T1D [OR (95% CI) = 0 (0–0.43), *P*c = 0.03]; this was also reported in the Tunisian (44, 45) and European populations (7, 9).

The HLA-DQA1 locus is understudied in most HLA-related association studies conducted in the Arab populations (27, 41, 42, 44, 45), which makes the four significant alleles identified in our study a boon for future comparative analyses. The most strongly associated allele within HLA-DQA1 was DQA1*05:01:01 [OR (95% CI) = 3.2 (2.05-4.92), *P*c = 3.0 x 10^-7^], which increased the risk of T1D. A similar observation has been reported in the Saudi Arabian (28) and Caucasian populations (7, 9). The other two significantly expressed alleles within HLA-DQA1, i.e., DQA1*05:05:01 [OR (95% CI) = 0.1 (0.02–0.34), *P*c = 9.7 x 10^-5^] and DQA1*01:03:01 [OR (95% CI) = 0.1 (0.01–0.39), *P*c = 0.001], showed protection against T1D, which has also been reported in Saudi Arabia (28). HLA-DQA1*03:01 [OR (95% CI) = 2.22 (1.23–4), *P*c = 0.04], another significantly expressed allele (2-fields analysis) identified in our study, is known to confer risk of T1D in the Kuwaiti (32) and Saudi populations (28).

In regard to the HLA-DQB1 locus, which has been studied in the Kuwaiti population (32), we identified the same allele, DQB1*02:01:01 [OR (95% CI) = 3.6 (2.3–5.66), *P*c = 2.2 x 10^-8^], to be strongly associated with the risk of developing T1D; this has also been reported in the Arab (27–29, 41–43), Korean (40), and Caucasian populations, and has been known to confer risk of T1D (7, 9). In our Kuwaiti population, another identified allele — DQB1*03:02:01 [OR (95% CI) = 3.1 (1.82–5.48), *P*c = 0.0001] was significantly associated with an increased risk of developing T1D; similar results have been reported in the Emirati (27), Saudi Arabian (28), Bahraini (41, 42), and European populations (7, 9). Lastly, we also identified DQB1*03:01:01 [OR (95% CI) = 0.1 (0.02–0.41), *P*c = 0.0009] as a significant allele that renders protection against T1D; similar observations were made by other researchers in the Arab region (27, 28, 41, 42). Interestingly, some alleles reported to be significant in association studies on HLA–T1D in the Arab populations were not identified in the present study. Nonetheless, these alleles might show the same distribution pattern across patients with T1D and controls, and/or they might be significant if the allele field is lowered in the analysis.

Expectedly, a combination of the significantly identified alleles within HLA-DRB1, HLA-DQA1, and HLA-DQB1 genes made it to the association analysis of haplotypes. Our data revealed that the strongest associated haplotype was DRB1*03:01:01~DQA1*05:01:01~DQB1*02:01:01 [OR (95% CI) = 4.1 (2.61–6.63), *P*c = 8.7 x 10^-10^], which confers high risk of T1D, and is well-documented in Arab, Korean, and Caucasian populations (7, 9, 40). Furthermore, DRB1*04:05:01~DQA1*03:03:01~DQB1*03:02:01 [OR (95% CI) = 5.4 (1.64–23.16), *P*c = 0.01] is the second-most significant haplotype conferring high risk of T1D and has only been reported once in a European population study (47). Enczmann et al., suggested that the identification of this haplotype is possible using high-resolution NGS, which was employed in our study, that genotyped both exon 2 and 3 of the DQ gene for allele classification. The third-most significant haplotype identified in our study (using 2 fields analysis) — HLA-DRB1*11:04-HLA-DQA1*05:05-HLA-DQB1*03:01 [OR (95% CI) = 0 (0–0.46), *P*c = 0.04] — demonstrated resistance to T1D, which, to the best of our knowledge, has not been reported previously.

The extent of zygosity at the significantly identified T1D risk haplotypes differed across the groups of age at onset. The DRB1*03:01:01~DQA1*05:01:01~DQB1*02:01:01 haplotype exhibited a higher frequency of homozygosity in the group of early age at onset, indicating that this haplotype in homozygous form confers a higher risk of developing T1D at an early age. Although DRB1*04:05:01~DQA1*03:03:01~DQB1*03:02:01 homozygous haplotype is seen less frequent in our cohort to draw conclusion, its rarity is uniformly seen across the three groups of age at onset.

The significant T1D risk haplotypes (namely DRB1*03:01~DQA1*05:01~DQB1*02:01 and DRB1*04:05~DQA1*03:03~DQB1*03:02) reported in this study are based on the Bonferroni-corrected P- values. Some of the other well-known haplotypes, reported in literature, with either T1D disease risk like DRB1*04:02~DQA1*03:01~DQB1*03:02 or protection against it like the DRB1*11:04~DQA1*05:05~DQB1*03:01, DRB1*13:01~DQA1*03:01~DQB1*06:03, DRB1*15:01~DQA1*01:02~DQB1*06:02 and DRB1*15:02~DQA1*01:03~DQB1*06:01 also appear in our study as significant albeit in uncorrected P values. It is possible that with increased cohort sizes in future studies, associations in haplotypes with low frequencies would be revealed. In addition, this study considers all the three fields of alleles in performing haplotype analysis. It may be pointed out that it is also possible to perform the analysis using only the first two fields since the significance of the third field remains unclear as the polymorphisms are not associated with amino acid changes and the field is very much the same in alleles defined by the first two fields.

Siblings of T1D children can exhibit increased risk for developing T1D risk. However, it is not possible to us to assess this as our study is not a long-term follow-up protocol. Nevertheless, we present results of longitudinal studies from literature on the subject. Generally, the overall risk of an individual developing T1D in a population is 0.4% (48). Nevertheless, the risk is higher for siblings of affected children (49). The estimated risk can significantly increase depending on the T1D proband’s age at onset, the presence of specific high-risk HLA alleles, and whether the siblings are monozygotic twins. For instance, siblings of T1D individuals with an early onset of less than 5 years have a higher cumulative risk of developing diabetes by age 20 years (11.7%), compared to 3.6% and 2.3% for those with onset between ages 5 and 9 years and between ages 10 and 14 years, respectively (50). In addition, sharing both HLA DR3/4-DQ8 haplotypes with a T1D proband elevates the risk of islet autoimmunity in siblings to 63% by age 7 and 85% by age 15, compared to those who do not share both haplotypes (20% by age 15). Of those sharing both haplotypes, 55% develop diabetes by age 12, compared to 5% without both haplotypes. Siblings without the HLA DR3/4-DQ8 genotype, despite carrying the same haplotypes with their T1D proband, had only a 25% risk of T1D by age 12 (51). Moreover, monozygotic twins are at higher risk (over 40%) of developing T1D and positive autoantibodies compared to non-twin siblings and dizygotic twins. Additionally, monozygotic twins with the HLA DQ8/DQ2 genotype have a greater risk of progressing to T1D and positive autoantibodies than those without (52).

Amino acid variations within the HLA genes and their association with T1D is understudied in Arab populations, as compared to studies on alleles and haplotypes. Although a modest attempt was carried out previously (32), with advancements in precise HLA genotyping techniques, such as NGS, the current study identified 66 amino acids positions that were significantly associated with T1D. In the present study, most of the significant amino acid positions either comprised protective or susceptibility attributes associated with T1D. Some of the significant amino acid positions identified on the HLA-DRB1 gene were previously reported in the Omani population, such as DRß1-11 and DRß1-71 (29), and the European populations, including DRß1-13, DRß1-70, DRß1-71, and DRß1-74 (10, 12, 37, 38). To the best of our knowledge, significant associations between T1D and changes in amino acids at position DRß1-26, DRß1-33, DRß1-37, DRß1-58, DRß1-67, DRß1-73, DRß1-96, DRß1-133, DRß1-140, DRß1-142, and DRß1-180, have not been reported previously, highlighting the novelty in our findings. Additionally, several amino acid changes that were significantly associated with T1D identified on the HLA-DQA1 gene have not been reported before, such as DQα1-11, DQα1-18, DQα1-26, DQα1-41, DQα1-45, DQα1-47, DQα1-48, DQα1-50, DQα1-52, DQα1-53, DQα1-56, DQα1-61, DQα1-64, DQα1-76, DQα1-80, DQα1-130, DQα1-175, DQα1-187, and DQα1-218. Whereas DQα1-55 and DQα1-66 positions, found to be associated with T1D, were reported in the Cypriot population (38). In addition, we uncovered novel and significant amino acid positions on the DQβ1 gene, such as DQβ1-13, DQβ1-28, DQβ1-37, DQβ1-45, DQβ1-46, DQβ1-52, DQβ1-53, DQβ1-55, DQβ1-66, DQβ1-67, DQβ1-71, DQβ1-74, DQβ1-84, DQβ1-85, DQβ1-86, DQβ1-87, DQβ1-89, DQβ1-90, DQβ1-125, DQβ1-167, DQβ1-203, DQβ1-220, and DQβ1-221. While amino acid positions, such as DQβ1-9, DQβ1-26, DQβ1-30, DQβ1-47, DQβ1-57, and DQβ1-70 have been previously reported to be associated with T1D in European populations (10, 13–15, 37–39). The identified amino acid positions that are significantly associated with T1D on the HLA-DRB1, HLA-DQA1, and HLA-DQB1 genes, whether previously reported or novel, might have a functional impact on the three-dimensional structure of the HLA genes, including antigen binding sites, and may either cause T1D or influence the age of T1D onset. Many of the significant amino acid positions that we identify are supported by many previous studies (at least 8 independent studies) as listed in the Results sections. However, it is to be noted that the observed amino acid variations have not been characterised for impact on the structural and functional features of the protein(s).

We additionally tested the prevalence of haplotypes predisposing to celiac disease and HT in our cohort. CD comprises only 0.02% of our T1D cohort; it is interesting to note that 47.4% carry DQA1*05:01/DQB1*02:01 encoding a DQ2.5 protein, which represents the strongest risk haplotypes associated with the celiac disease and additionally shared by T1D (53, 54). Similarly, HLA DR3-DQ2.5 and DR4-DQ8 are the major risk haplotypes associated with T1D in our study. More than 90% of the patients with celiac disease are reported to carry HLA DR3–DQ2.5 haplotype (55). Certain common predisposing alleles specifically DQB1*02:01:01 and HLA DQA1*05:01:01 are observed in significantly increased frequency in our T1D cohort compared to controls (**Table 1**). Though autoimmune thyroid conditions such as HT and Graves’ disease are recurrently associated with T1D, only one of the forms namely Hashimoto’s was detected in our cohort at a frequency of 0.03%. Limited studies have investigated the link between HLA class II alleles and HT, DR3 and DR4 haplotypes are the common haplotypes associated with the disease (56, 57). Each of autoimmune condition can co-exist with T1D especially if they have the same high-risk HLA profile, nevertheless, the diagnosis of one does not necessarily imply the presence of others especially at the same time. In our study T1D cohort we have limited number of individuals with CD and HT which highlight the complex multi-factorial nature of the autoimmune disorders.

In summary, our findings contribute to the growing body of knowledge about the genetic factors influencing the risk of developing T1D in children. This information has clinical implications for diagnosis, risk assessment, and personalized management of T1D, which can ultimately help improve the lives of affected individuals and their families.

In our current study, we utilized a higher typing resolution to investigate the association between the classical HLA class II genes and T1D in the Kuwaiti population. This approach allowed us to examine amino acid variations that were not explored in previous studies conducted in Kuwait (32, 33, 46). Furthermore, most T1D studies in Arab populations, with the exception of one conducted in Saudi Arabia (28), have allele resolutions ranging from 1 to 2 fields. This variation in resolution may potentially impact the overall association results (27, 29, 42–45). Furthermore, this study provides several novel results that may offer great clinical and research benefits. Despite these strengths, the results of our study come with few limitations. First, the sample size of people with T1D is relatively small even though its larger than prior studies performed in Kuwaiti population (32). Nevertheless, a larger sample size may provide a comprehensive portfolio of variations in allele, haplotype, and amino acid frequencies and allow association tests within specific T1D-related alleles (13, 14). Second, we carefully screened our control group to exclude any individuals with a family history of T1D or symptoms suggestive of adult-onset T1D. While our control group had a higher proportion of females than males, genomic autosomal HLA risk haplotypes do not generally differ based on sex (58). However, there is suggestive evidence for existence of sex-dependent differences in islet autoimmunity for T1D high-risk haplotypes (59), which we acknowledge as a potential confounder which could not be addressed in our study due to non-availability of full autoimmunity profiles of the study participants. Lastly, we cannot rule out mistype alleles resulting from algorithmic error by the HLA typing software, as this has been reported in other HLA typing tools such HLAforest, HLAminer, and PHLAT (60).

## Conclusion

The significant findings on the association between alleles, haplotypes, and amino acid variations and T1D in the Kuwaiti population are not far from what has been previously reported in the Arab and European populations. Moreover, we further uncovered novel haplotypes and amino acid positions within HLA class II genes that are associated with T1D, which may shed some light on the understanding of immunogenetic influences on T1D.

## Data availability statement

The datasets presented in this article are not readily available because they pertain to minor children. Requests to access the datasets should be directed to either FA-M (fahd.almulla@dasmaninstitute.org) or TT (alphonse.thanaraj@dasmaninstitute.org).

## Ethics statement

The studies involving humans were approved by Ethical Review Committee of Dasman Diabetes Institute. The studies were conducted in accordance with the local legislation and institutional requirements. Written informed consent for participation in this study was provided by the participants’ legal guardians/next of kin.

## Author contributions

Study design: MD, TT, FA-M. Clinical recruitment: HA and EO. Whole exome sequencing and targeted HLA typing: RN and SJ. Genetic and statistical analysis: MD. Manuscript writing: MD, TT, and RN. Critical review of the manuscript: EO, TT, and FA-M. All the authors approved the manuscript. All authors contributed to the article.

## Supplementary material

Supplementary Table 1 Results of the associations between the HLA-DRB1, HLA-DQA1, HLA-DQB1 genes and children with T1D or controls.

Supplementary Table 2 Distribution of DRB1, DQA1, and DQB1 amino acid residue variations among children with T1D and controls.
